# Comparing the cost effectiveness of harm reduction strategies: a case study of the Ukraine

**DOI:** 10.1186/1478-7547-12-25

**Published:** 2014-11-24

**Authors:** Sung Wook Kim, Anni-Maria Pulkki-Brannstrom, Jolene Skordis-Worrall

**Affiliations:** UCL Institute for Global Health, 30 Guilford Street, London, WC1N 1EH UK; Epidemiology and Global Health Department, Umeå University, Umea, Sweden; London School of Hygiene and Tropical Medicine, Keppel Street, London, WC1E 7HT UK

**Keywords:** Harm reduction, Cost effectiveness analysis, Needle and syringe programme, Opioid substitution therapy, Ukraine, Markov Monte Carlo simulation, Global Fund

## Abstract

**Background:**

Harm reduction strategies commonly include needle and syringe programmes (NSP), opioid substitution therapy (OST) and interventions combining these two strategies. Despite the proven effectiveness of harm-reduction strategies in reducing human immunodeficiency virus (HIV) infection among injecting drug users (IDUs), no study has compared the cost-effectiveness of these interventions, nor the incremental cost effectiveness of combined therapy. Using data from the Global Fund, this study compares the cost-effectiveness of harm reduction strategies in Eastern Europe and Central Asia, using the Ukraine as a case study.

**Methods:**

A Markov Monte Carlo simulation is carried out using parameters from the literature and cost data from the Global Fund. Effectiveness is presented as both QALYs and infections averted. Costs are measured in 2011 US dollars.

**Results:**

The Markov Monte Carlo simulation estimates the cost-effectiveness ratio per infection averted as $487.4 [95% CI: 488.47-486.35] in NSP and $1145.9 [95% CI: 1143.39-1148.43] in OST. Combined intervention is more costly but more effective than the alternative strategies with a cost effectiveness ratio of $851.6[95% CI: 849.82-853.55].

The ICER of the combined strategy is $1086.9/QALY [95% CI: 1077.76:1096.24] compared with NSP, and $461.0/infection averted [95% CI: 452.98:469.04] compared with OST. These results are consistent with previous studies.

**Conclusions:**

Despite the inherent limitations of retrospective data, this study provides evidence that harm-reduction interventions are a cost-effective way to reduce HIV prevalence. More research on into cost effectiveness in different settings, and the availability of fiscal space for government uptake of programmes, is required.

## Background

There are an estimated 15.9 million injecting drug users (IDUs) worldwide, 80% of whom live in developing and transitional countries [1]. The concurrent epidemics of HIV and injecting drug use have rapidly increased HIV prevalence [2], with 10% of HIV/Acquired Immunodeficiency Syndrome (AIDS) cases worldwide attributed to IDUs [3, 4].

HIV prevalence in Eastern Europe and Central Asia has almost tripled since 2000 [1], to an estimated 1.4 million people in 2011 [5]. The region is also home to 3.7 million IDUs [1]. Ukraine’s HIV prevalence is the highest in Europe and a 2010 study found that 50% of IDUs in Ukraine were HIV positive [6]. The Global Fund has spent approximately $20 m on harm reduction in the Ukraine [7].

Harm reduction interventions aim to reduce the harmful results of drug use. Although there is no agreed definition, a small number of interventions are commonly described in the literature as “harm reduction” including; condom provision, community based outreach, peer-led interventions, needle and syringe programmes (NSP) and opioid substitution therapy (OST) [8]. Needle and syringe programmes offer a clean needle and syringe to injecting drug users (IDUs), while opioid substitution therapy (OST) replaces heroin with a less addictive drug such as methadone or buprenorphine under medical supervision. As IDUs undergoing OST may continue drug use outside of the programme [9], combining NSP with OST may be more effective in reducing HIV transmission than a single intervention [10]. This paper will focus only on three harm reduction strategies; NSP, OST, and combined therapy (NSP and OST) based on the literature review.

This study’s purpose is to improve our understanding of the relative cost effectiveness of harm reduction strategies. A literature review on the cost-effectiveness of harm reduction describes what is known, and what is not. A cost-effectiveness analysis of harm-reduction is then conducted using data from the Global Fund for the Ukraine. The analysis compares NSP, OST and a combined intervention. To the best of our knowledge, this is the first study comparing a combined intervention with NSP or OST alone, in any setting.

## Literature review

The literature on harm reduction cost-effectiveness was reviewed to summarize current evidence. Web of Science, Econlit and Pubmed were the primary databases searched. A supplementary search was conducted using Google Scholar. Reference lists of identified papers were hand-searched for further appropriate papers. The search terms were: cost-effectiveness, HIV, NSP, OST, harm reduction, needle and syringe, and methadone. Only papers published in English, in peer-reviewed journals were considered.

The inclusion criteria were: 1) Studies should be about harm reduction interventions i.e. NSP, OST, or combined therapy; 2) Studies should be analyzed from a health economic perspective 3) Studies should present data on either effectiveness or cost, and 4) Studies should focus on HIV. 5) Papers not available in English or published before 1990 were excluded. The initial search identified 18 papers. After reviewing titles and abstracts, 3 were excluded. After reading the full papers, 3 further papers were excluded and 3 were added following a hand search of the reference lists. The final review includes 15 papers listed in Table 1, two of which are literature reviews themselves.

**Table 1:** **Summary of systematic review result**

Study	Comparator	Intervention Evaluated	Form of economic analyses	Perspective taken	Model used	Time horizon	Outcome measure
Jones et al. [11]†			Cost utility (N = 12) Cost benefit (N = 1)		Behavioural models using simplified Bernoulli process (N = 4) simulated the transmission (N = 2) the theory of needle circulation originally developed by Kaplan and O’Keefe (N = 4)		HIV incidence (N = 11) HCV incidence (N = 1) HIV and HCV incidence (N = 1)
Connock et al. [12]†			Cost–utility (N = 5)	Societal perspective (N = 5) healthcare system (N = 6)	Markov (N = 3) Dynamic (N = 3) Monte Carlo (N = 1)		QALY (N = 6)
Belani and Muennig [13]	no NSP	NSP	Cost utility	Societal	Decision model	1 year	Infection averted & QALYs
Wammes et al. [14]	OST	OST (coverage 5% to 40%)	Cost effectiveness/cost analysis	Societal	Mathematical transmission model	20 year	Infection averted
Guinness et al. [15]	non intervention	OST	Cost utility	Provider	Mathematical model	3 year	Infection averted & DALYs
Tran, Mills, et al. [16]	non OST	OST	Cost utility	Health service provider	Real cohort data	9 month	QALYs
Tran, Nguyen, et al. [17]	non OST	OST	cost utility	Vietnam health care system	Decision analytical model	1 year	Infection averted & QALYs
Tran, Ohinmaa, et al. [18]	OST&ART	OST	Cost utility	Vietnam health care system	Decision tree monte carlo simulation	1 year	Infection averted & QALYs
Connock et al.[12]**	Buprenorphine	OST (methadone vs buprenorphine)	Cost utility	NHS	Markov monte carlo simulation	1 year	QALYs
Degenhardt et al. [10]	ART, NSP&OST&ART	Combined (NSP&OST)	Cost effectiveness		Transmission model	5 years	Infection aveted
Alistar, Owens, and Brandeau [19]	OST&ART ART alone	OST	Cost utility	Provider	dynamic compartmental model	20 year	Infection averted & QALYs
Li et al. [20]	ART, VCT	Combined (NSP&OST)	Cost utility		Mathematical	30 year	Infection averted & QALYs
Van den berg et al.* [21]	Methadone dose or NEP use alone	Combined (NSP& OST)		Not stated	Cohort study	20 years	Incidence rate ratio
Kwon et al. [22]	no NSP	NSP	Cost utility	Health sector	Mathematical model	lifetime	Infection averted & QALYs
Zhang et al. [23]	no NSP	NSP	Cost utility	Societal	Mathematical model	7 years	Infection averted & DALYs

†Systematic review.

*included in Jones et al. [11].

**Connock et al. [12] carried out a systematic review and a cost effectiveness analysis in one paper.

This literature review summarizes the systematic review papers [11, 12] and then lists the papers published since their publication [10, 13–17, 19, 20, 22–24]. One exceptional case is the research by Van den berg et al. [21]. This study was added to bolster the evidence around the combined intervention because the majority of the papers since the systematic reviews focus on a single therapy. Only one retrieved study by Degenhardt et al. [10] focused on the effectiveness of combined intervention of NSP and OST.

The first two papers in Table 1 are reviews of harm reduction strategies by Connock et al. [12] and Jones et al. [11]. Connock et al. [12] conducted a systematic review and cost effectiveness analysis of OST. They conclude that methadone dominates buprenorphine, both of which are licensed for use as opioid substitutes. Jones et al. [11] conducted a systematic review of NSP. Jones et al. [11] conclude from their review that in terms of reducing HIV incidence and prevalence among IDUs, NSPs are cost-effective.

While the review papers aimed to explore the cost effectiveness of a single intervention, a number of studies conducted since the reviews, have attempted to compare these single interventions with an alternative. For example, Van den berg et al. [21], (cited in the review by Jones et al.) compared a combined intervention with incomplete harm reduction. They concluded that combined intervention is more cost effective than incomplete harm reduction. Van den berg et al. [13] compared full harm reduction (NSP + OST) vs incomplete harm reduction (NSP + OST). However, they assumed incomplete harm reduction always offers OST, just changing ‘the dose of OST’. Therefore, patients who get incomplete harm reduction always get OST as a base case. Likewise, Degenhardt et al. [10] compared combined intervention (OST + NSP) with ART and found that combined intervention of OST and NSP and ART gained more effectiveness than either OST + NSP or ART.

All NSP studies that reported NSP as a primary intervention used ‘no NSP and no intervention’ as a comparator. However, studies of OST show more varied comparators; ‘no OST’ [15–17], combined ART intervention [10, 20], and buprenorphine [12].

Of the papers listed in Table 1, ten used either QALYs gained or DALYs averted as a measure of outcome. All of the papers estimated both cost per infection averted and cost per either QALY or DALY.

In general, these studies suggest that harm reduction interventions are cost effective and particular reference is made to OST [18, 19] and NSP [22, 23]. These results are derived mainly from a mathematical and epidemiological modelling [14, 15, 22, 23, 25] rather than from cohort studies [21]. This review also highlights a gap in the evidence regarding the relative cost effectiveness of harm reduction strategies. It is necessary to compare which mono-therapy (OST or NSP) is more cost effective, and whether mono-therapy is more cost effective than combined intervention – whether or not ART is offered. This is not because the provision of ART is unimportant, but because the literature has moved on to evaluate the cost effectiveness of the provision of ART with harm reduction – without first considering what is the most cost effective harm reduction package. As described above, there are studies arguing for the relative cost effective of NSP [13], of OST [14] and of combined intervention [10, 20, 21]. However, no study that we could find compared the cost effectiveness of these three harm reduction alternatives. To fill this evidence gap, we conduct a CEA of harm reduction comparing NSP, OST, and combined intervention, using each intervention as a comparator. Exclusion of ART from harm reduction interventions appears to be feasible, considering the low ART coverage among IDUs in the Ukraine, which was 26.7% in 2004 and 24% in 2008 [26, 27].

### Section 2: case study of the Ukraine

The largest IDU populations in Eurasia are in the Russian Federation (1.8 million) and the Ukraine (296,000) [1]. Nearly half of IDUs in the Ukraine live with HIV [1], the highest prevalence rate in Europe [6]. The Global Fund has spent approximately $ 20 m on harm reduction in the Ukraine since 2004 [7]. Considering that the Global Fund disbursed US$ 361 million through 120 grants in 55 countries between 2002 and 2009 [28], the amount spent in the Ukraine is significant for a single country. That said, while the Global Fund provides significant international support for harm-reduction programs [2, 28], these investments have seldom been evaluated.

## Methods

Consistent with previous research in this area [12, 29], this study uses a Markov model, assuming three states of ‘infected’, ‘uninfected (well)’, and ‘dropped’. The model is designed to estimate costs and outcomes in terms of QALYs and HIV infections averted over 60 months, for the three strategies. The model estimates uncertainty using probability distributions. Monte Carlo simulation was then carried out using these distributions to account for uncertainty in the results of the model.

**Figure 1:**
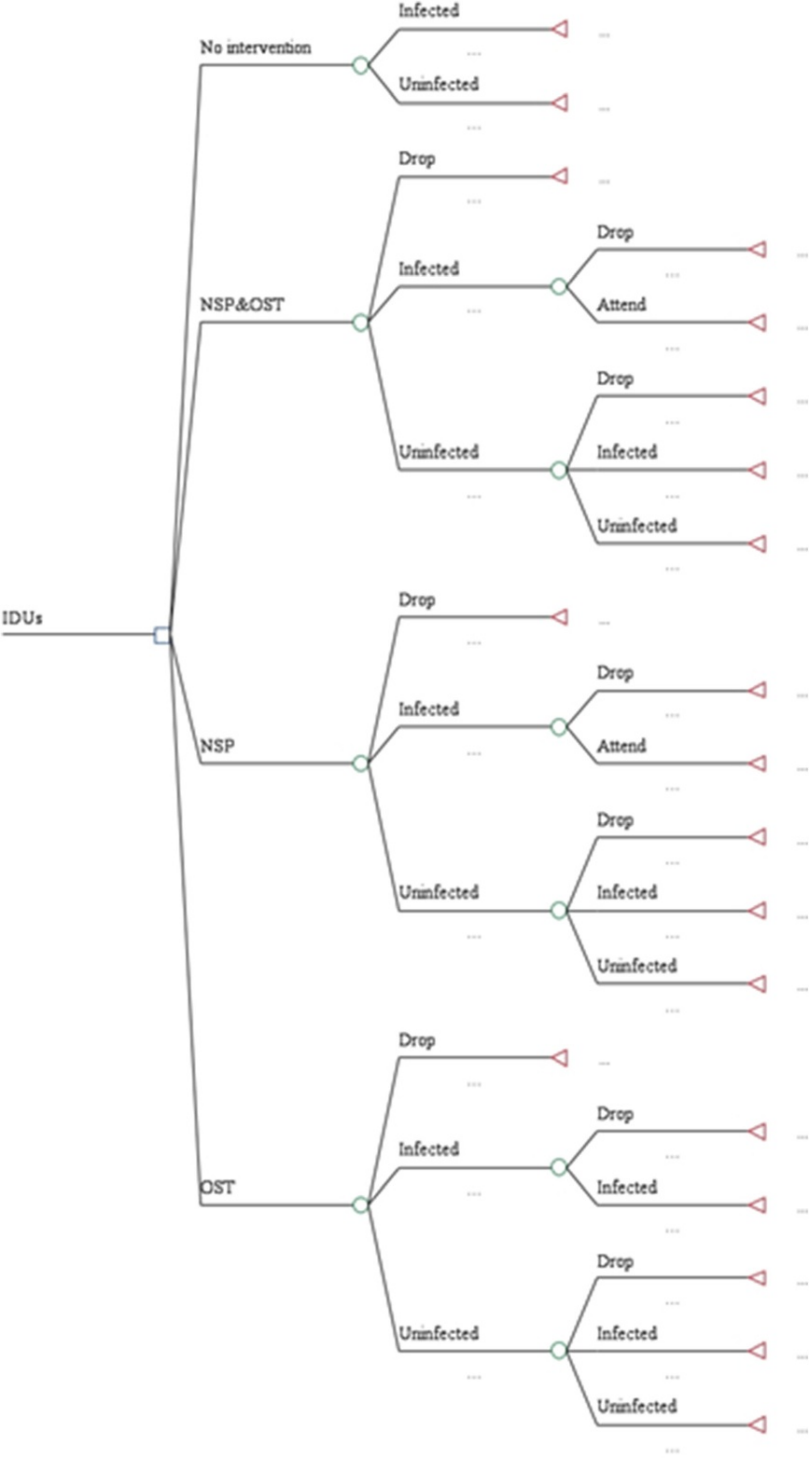
**Decision model.**

Figure 1 illustrates one cycle of the Markov decision model in this study. ‘Infected’ status occurs when patients are confirmed as HIV positive while ‘uninfected’ occurs when patients are HIV negative. ‘Dropped’ means that IDUs quit any harm reduction interventions they were attending. It is assumed that there is no mortality within the 5 year harm reduction period based on the existing literature (Table 2). This assumption is consistent with the approach used by Vickerman et al. [25] (Table 2). As the objective of this study is to compare the cost effectiveness of harm reduction strategies irrespective of ART provision, ART is not offered in any intervention including ‘no intervention’.

**Table 2:** **Model parameters**

Simulation parameters				
**HIV incidence**	Base case value	Duration	Distribution	Source
Number of injections	400.00	1 year		Degenhardt et al. [10] Aceijas et al. [3]
reduction in frequency of drug injections per day from the cohort data	0.85	1 year		Alistar, Owens, and Brandeau [19]
Pr of transmission	0.01	1 year		Gouws et al. [30]
using sterile injection equipment or condom or methadone	0.90	1 year		Assumed based on Cao et al. [31]; Vickerman et al. [25]; Alistar et al. [19]
number of days follow up	60 cycle(5 years)	60 months(treatment)		Global Fund [7]
HIV prevalence among IDUs(NSP)	0.43	1 year		Calculated using Vickerman et al. [25]
HIV prevalence among IDUs(OST)	0.28	1 year		Calculated using Vickerman et al. [25]; Alistar, Owens, and Brandeau [19]
HIV prevalence among IDUs(NSP&OST)	0.18	1 year		Calculated using Degenhardt et al. [10]
Decrease in HIV incidence(NSP)	0.22	1 year		Vickerman et al. [25]
Decrease in HIV incidence (OST)	0.53	1 year		Alistar, Owens, and Brandeau [19]
Decrease in HIV incidence (NSP&OST)	0.66	1 year		Degenhardt et al. [10]
**Probability**				
Pr(attend to intervention)	0.0750	1 cycle	Beta	Assumed
Pr(mortality) if no intervention	0.03	1 cycle	Beta	Vickerman et al. [25]
Pr(infected) if no intervention	0.0446	1 cycle	Beta	Vickerman et al. [25]
Pr(well) if no intervention	0.0388	1 cycle	Beta	calculated
Pr(drop) from NSP&OST	0.0083	1 cycle	Beta	Calculated Pr(attend to intervention)
Pr(infected) from NSP&OST	0.0003	1 cycle	Beta	Degenhardt et al. [10]
Pr(well) from NSP&OST	0.9914	1 cycle	Beta	Calculated
Pr(drop) from NSP	0.0083	1 cycle	Beta	Calculated Pr(attend to intervention)
Pr(infected) from NSP	0.0005	1 cycle	Beta	Vickerman et al. [25]
Pr(well) from NSP	0.9912	1 cycle	Beta	Calculated
Pr(drop) from OST	0.0083	1 cycle	Beta	Calculated Pr(attend to intervention)
Pr(infected) from OST	0.0004	1 cycle	Beta	Alistar, Owens, and Brandeau[19]
Pr(well) from OST	0.9913	1 cycle	Beta	Calculated
Pr(infected) if dropped from intervention	0.0446	1 cycle	Beta	Vickerman et al. [25]
Pr(well) if dropped from intervention	0.9554	1 cycle	Beta	Calculated
Pr(drop) if infected from NSP&OST	0.0102	1 cycle	Beta	Yin et al. [32]; Jones et al. [11]
Pr(attend) if infected from NSP&OST	0.9898	1 cycle	Beta	Calculated
Pr(drop) if infected from NSP	0.0196	1 cycle	Beta	Jones et al. [11]
Pr(attend) if infected from NSP	0.9804	1 cycle	Beta	Calculated
Pr(drop) if infected from OST	0.0433	1 cycle	Beta	Yin et al. [32]
Pr(well) if infected from OST	0.9567	1 cycle	Beta	Calculated
**QOL**				
NSP	0.85		Normal	Vickerman et al. [33]
OST	0.74(average value for 54 week)		Normal	Connock et al. [12]
NSP & OST	0.95		Normal	Vickerman et al. [33]
Infected(dropped)	0.63		Normal	Connock et al. [12]
**Cost**				
NSP				
unit cost per patient	151.14	1 year	gamma	Global Fund [7]
Fixed	1197008.80	1 year	gamma	Estimated from the Global Fund grant proposal
OST				
unit cost per patient	1752.00	1 year	gamma	WHO medical database
Fixed	700050.60	1 year	gamma	Estimated from the Global Fund grant proposal
NSP&OST				
unit cost per patient(per year)	1903.14	1 year	gamma	Global Fund [7], WHO medical database
Fixed	168286.96	1 year	gamma	Assumed from the Global Fund grant proposal
**Other parameters**				
Consumer price index(CPI)	1.5680			WorldBank [34]
Time horizon	5 years			WHO [35]
Discount rate for cost	0.03		Uniform	WHO [35]
Discount rate for outcome	0.03		Uniform	WHO [35]
Population(NSP)	11000.00			Global Fund [7]
Population(OST)	5000.00			Global Fund [7]
Population(NSP & OST)	6000.00			Global Fund [7]
Initial HIV prevalence among IDUs	0.53	1 year		Vickerman et al. [25]
IDU mortality rate per 1000 person-years	0.4	1 year		Vickerman et al. [25]

A Markov Monte Carlo simulation with 10,000 iterations was conducted. It is known that at least 440 iterations should be run to be 95% sure that the estimate of the mean of the output is accurate [36]. Consequently, it can be said that 10,000 iterations are sufficient to get a 95% confidence interval. This figure of 10,000 iterations is consistent with other research on HIV [37, 38] and is recommended for medical decision making generally [39]. The main outcomes are expressed as cost effectiveness ratios (CE) and incremental cost-effectiveness ratios (ICERs).

Treeage software was used to construct the model. Sensitivity analysis was carried out to account for uncertainty in the cost data. Potential affordability of the three strategies was assessed using probability sensitivity analysis (PSA) with the GDP of the Ukraine as a threshold. Based on WHO recommendations, an intervention may be considered cost effective if the cost per QALY is less than the country’s GDP per capita [35, 40]

### Interventions compared



**Figure 2:**
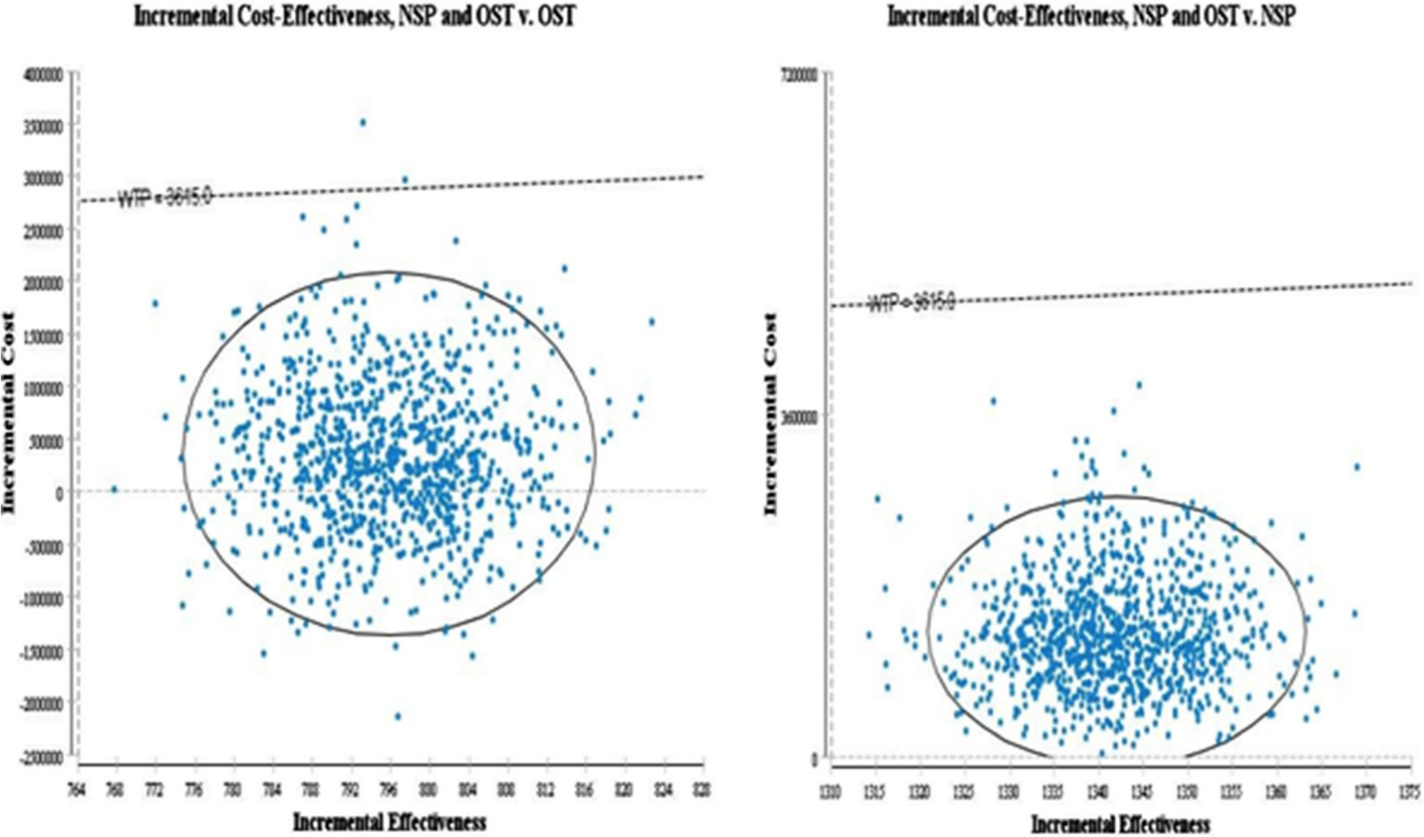
**Probabilistic sensitivity analysis(Y axis: Incremental cost X axis: HIV infection averted, Dotted line: WTP = GDP per capita of the Ukraine).**

**Figure 3:**
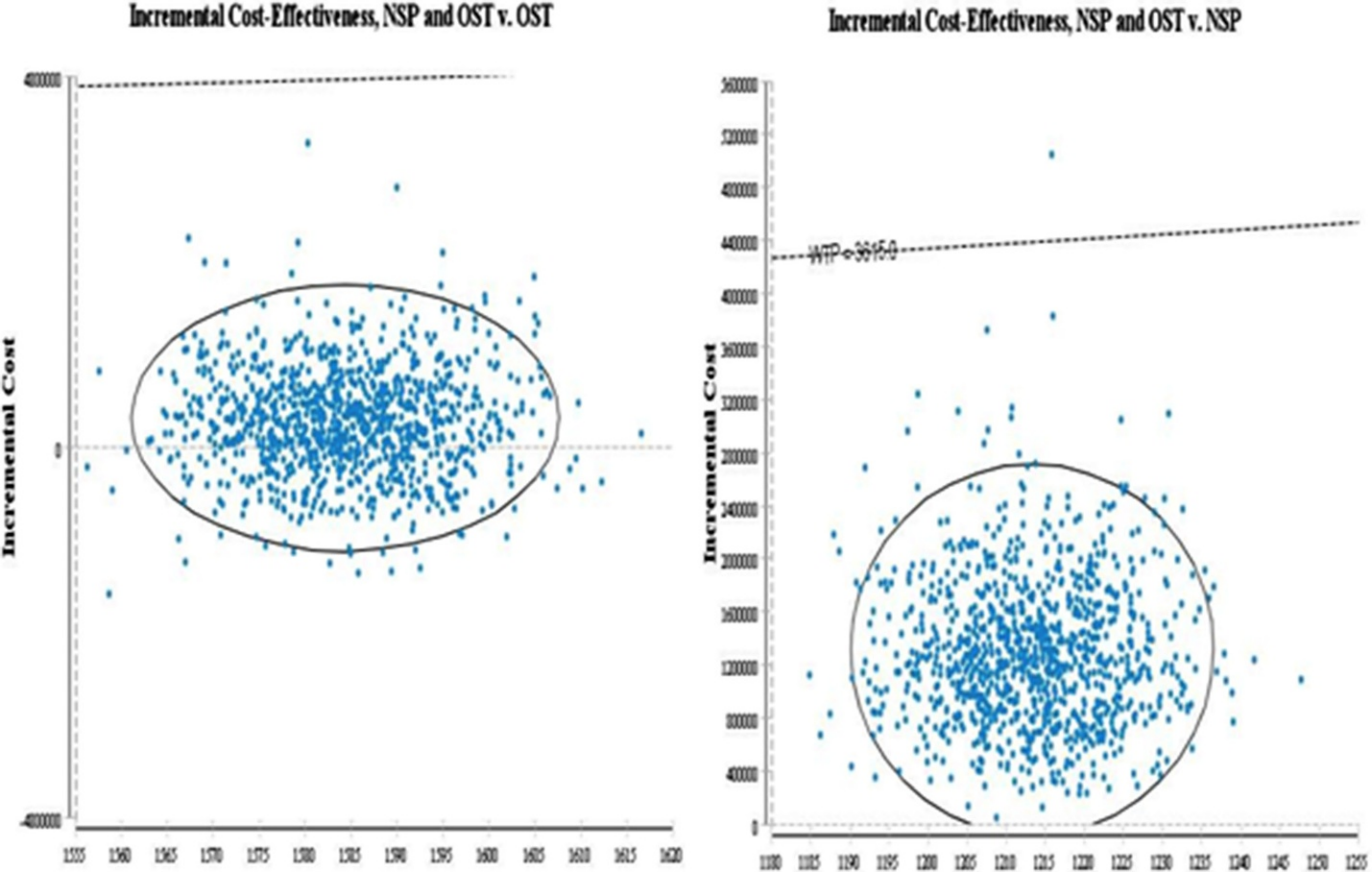
**Probabilistic sensitivity analysis(Y axis: Incremental cost X axis: QALYs, Dotted line WTP = GDP of the Ukraine).**

C1 is the cost of the new intervention, and E1 is the effect of the new intervention, whereas C0 and E0 are the cost and effect of the base case or comparator. Six cases were considered in this study: NSP vs OST, NSP vs NSP&OST, NSP vs no intervention, OST vs NSP&OST, OST vs no intervention, NSP&OST vs no intervention. The result is shown as a form of PSA in Figures 2 and 3. The base case is a no intervention, in which IDUs do not get any harm reduction intervention.

HIV incidence was obtained from previous literature. Given the initial HIV prevalence [25], the effectiveness of each intervention was estimated. HIV incidence and infections averted, were calculated with the following formula [16, 30]:


where S = total no of susceptible individuals (IDUs)

*α* = reduction in frequency of drug injections per day from the cohort data

*β* = Pr of transmission

*γ*= reported using sterile injection equipment or condom or methadone

n = number of days follow up

p = HIV prevalence among IDUs

It was assumed that IDUs inject needles and syringes 400 times per year [10, 41] and that they inject constantly for the entire program period. The HIV prevalence rate of IDUs for each intervention were calculated using parameters from different papers [10, 19, 25], assuming that they are already getting each intervention as data was not available for HIV prevalence rate. The values of alpha and gamma are shown as a proportion, and corresponding parameter values are presented in Table 2.

### Distribution of parameters

To implement Monte Carlo simulation, the costs of each intervention were estimated based on gamma distribution, which is nonnegative and allows the maximum likelihood estimate of the population mean to be the sample mean (Table 2) [42]. The probability distribution of each intervention was estimated with beta distribution, the value of which is between 0 and 1. Normal distribution was used for utilities of outcome for interventions. For the discount rate, a uniform distribution between 0 and 1 was used.

### Probability

In this study, transition probabilities for the ‘infected’, ’uninfected (well)’, and ‘dropped’ state are shown in Table 2. QALYs for each state were obtained from existing research [12, 25].

Cycle times of 1 month or 1 year are generally used in Markov model for chronic diseases [43]. Although 1 year can be used for HIV [44], IDUs can drop out of the interventions sooner. Therefore, a cycle length of 1 month is more appropriate for this model. Since each cycle in the model is 1 month, these transition probabilities were adjusted for a monthly base.

The prevalence rates of each intervention were obtained from previous research that calculated the rates considering needle sharing between IDUs, condom use and sexual behaviours [25]. The dropout rates at each cycle, for each intervention, were obtained from existing research [11, 32] (Table 2).

### Costs

Cost data were collected from the Global Fund website (http://www.theglobalfund.org). The Global Fund’s intervention in the Ukraine includes various harm-reduction packages [28]. In addition, other interventions were carried out simultaneously including public health education campaigns around harm reduction for IDUs, and other complementary activities. As a result, the exact proportion allocated to the combined intervention is not clear. For the purposes of this analysis then, it was conservatively assumed that the variable cost of the combined strategy is simply the sum of the variable costs for NSP and OST. Alistar et al. [19] calculated the cost of combined intervention of OST and ART in this way. They assumed identical staff costs such as counselling costs for both interventions and that the costs of the combined intervention are the sum of the variable costs for OST and ART. Staff costs and other delivery costs were included in start-up costs for each cycle. The variable costs of each intervention were annualised. Indirect costs, such as productivity loss, were not taken into account in this study and noted as a limitation of this analysis. Budgets for Round 6 (2006–2010) were also used for the combined intervention, although OST alone and the combined intervention was simultaneously implemented. This limitation in the cost data will be tested with sensitivity analysis.

The summarized grant data for the Ukraine is presented in Table 3-a. Costs per cycle were estimated based on the summarized data in Table 3-b, which describe how the harm reduction interventions of NSP and OST were conducted for 5 years. The variable cost of NSP for each cycle in the Ukraine comprises disposable syringes, needles, disinfectant solutions, and alcohol wipes [7]. The variable cost of OST is from the WHO medical database, using the price from Pharmascience.inc [7].

**Table 3:** **Cost data**

**a) Grant data Summary**							
	**Intervention**	**Year 1**	**Year 2**	**Year 3**	**Year 4**	**Year 5**	**Total**
Ukraine	harm reduction_syringe	522,600	1,372,411	1,901,061	1,900,411	1,951,061	7,647,544
	Substitution therapy	1,127,125	1,443,544	3,099,845	3,194,531	3,395,208	12,260,253
**b) Cost per cycle**							
**Cost per 1 cycle**		NSP		OST		Combined	
Starting up cost		99750.73		58337.55		14023.91	
Cost per patient		2.52		29.20		31.72	
**Total cost per 1 cycle**							
Cost per patient		11.59		40.87		34.06	

*All figures in table 3 is USD.

Costs were incurred in United States dollars (USD) and adjusted for inflation to 2011 values using the consumer price index (CPI) for the Ukraine from the World Bank [45]. All costs other than the variable costs of NSP and OST were classified as start-up costs (Table 3-b), which are generated at each Markov cycle of 1 month irrespective of the number of patients. The information regarding the total number of patients to calculate a cost per patient was obtained from the Global Fund website [46] and is presented in Table 2.

## Discounting, perspective and time horizon

The intervention’s benefits were evaluated over the duration of the grant period i.e. from the start of 2002 to the end of 2006. Annualized costs were used for each year. As the World Health Organisation (WHO) recommended, an identical discount rate of 3% was applied to both costs and effectiveness. A provider perspective was applied.

## Population

Using data from the literature, it was assumed that half of IDUs attending the interventions were infected at the outset [1, 25] (Table 2) and the average age was assumed to be 39 [47].

## Results

The results of the Markov Monte Carlo simulation are given with 95% confidence intervals in Table 4. The result of costs and effectiveness of deterministic analysis is located within the confidence interval of probabilistic analysis, showing the robustness of the result regarding the parameters for this model.

**Table 4:** **10,000 times Monte Carlo simulation results and a deterministic result of cost and effectiveness**

Infection averted		Monte Carlo Simulation			Deterministic		
		Combined	NSP	OST	Combined	NSP	OST
Cost		1,574,559.00	247,108.68	1,206,760.80	1,565,967.17	247,148.20	1,199,134.00
	Std	580,415.89	67,045.82	419,480.75			
	Upper CI(95%)	1,585,935.20	248,422.78	1,214,982.60			
	Lower CI(95%)	1,563,182.90	245,794.58	1,198,539.00			
Effect		1,848.76	506.98	1,053.10	1,848.74	506.98	1,053.03
	Std	6.78	5.33	5.39			
	Upper CI(95%)	1,848.89	507.08	1,053.21			
	Lower CI(95%)	1,848.63	506.88	1,052.99			
CE		851.68	487.41	1,145.91	847.05	487.49	1,138.75
	Std	95.06	54.01	128.57			
	Upper CI(95%)	853.55	488.47	1,148.43			
	Lower CI(95%)	849.82	486.35	1,143.39			
**QALY**							
		combined	NSP	OST	combined	NSP	OST
Cost		1,563,571.10	247,577.10	1,195,319.80	1,565,967.17	247,148.20	1,199,134.00
	Std	579,705.31	66,864.85	418,960.03			
	Upper CI(95%)	1,574,933.30	248,887.65	1,203,531.40			
	Lower CI(95%)	1,552,208.90	246,266.55	1,187,108.10			
Effect		4,183.51	2,970.21	2,599.08	4,183.51	2,970.21	2,599.04
	Std	7.47	5.69	5.71			
	Upper CI(95%)	4,183.66	2,970.32	2,599.19			
	Lower CI(95%)	4,183.36	2,970.10	2,598.97			
CE		373.75	83.35	459.90	374.32	83.21	461.38
	Std	95.06	54.01	128.57			
	Upper CI (95%)	374.57	83.55	460.93			
	Lower CI (95%)	372.92	83.15	458.87			

Combined therapy of NSP and OST averted the most infections (1848 HIV infections averted). After this, OST alone averted the most infections (1053 HIV infections averted). Combined therapy averted more infections than the sum of OST alone and NSP alone (1848 HIV infection averted vs 1559 HIV infection averted). Considering QALY gains, combined therapy still gained most (4183.5 QALYs). After this, NSP alone gained slightly more QALYs than OST alone (2970 QALYs vs 2599 QALYs).

Although combined therapy strictly dominated in terms of benefits, NSP alone was most cost effective at $487.4/infection averted and $83.3/QALY gained compared with combined therapy of NSP and OST together at $851.6/infection averted and $373.7/QALYs (Table 4). OST alone had the highest cost effectiveness ratios at $1145.9/infection averted and $459.9/QALYs.

### Sensitivity analysis

The costs of combined NSP and OST are uncertain due to the limitations of the cost data, which do not explicitly state the total costs of the combined intervention. Consequently, one-way sensitivity analysis was conducted to relax this limitation. The uncertainty of both effectiveness and costs was examined using PSA. The three strategies were compared with a single therapy in Table 5.

**Table 5:** **Sensitivity analysis**

Averted		Lower end	ICER	CI (95%)	Higher End	ICER	CI (95%)	Comparator
Variable_NSP	No intervention	0.00	0.00		2.52	0.00		
Variable_NSP	NSP	0.00	296.71	293.40:298.60	2.52	487.49	484.89:490.09	vs no intervention
Variable_NSP	OST	0.00	1920.53	1911.68:1929.38	2.52	1743.40	1734.55:1752.25	vs NSP
Variable_NSP	NSP and OST	0.00	461.01	452.98:469.04	2.52	461.01	452.98:469.04	vs OST
Variable_OST	No intervention	0.00	0.00		29.20	0.00		
Variable_OST	OST	0.00	83.17	82.73:83.61	29.20	1743.40	1734.55:1752.25	vs NSP
Variable_OST	NSP	0.00	−292.22	(−294.6): (−289.4)	29.20	487.49	484.89:490.09	vs no intervention
Variable_OST	NSP and OST	0.00	1857.95		29.20	461.01	452.98:469.04	vs OST
Startingcost_NSP	No intervention	0.00	0.00		99750.75	0.00		
Startingcost_NSP	NSP	0.00	190.78	187.4:192.6	99750.75	487.49	484.89:490.09	vs no intervention
Startingcost_NSP	OST	0.00	2018.89	2009.95:2027.65	99750.75	1743.40	1734.55:1752.25	vs NSP
Startingcost_NSP	NSP and OST	0.00	461.01	452.98:469.04	99750.75	461.01	452.98:469.04	vs OST
Startingcost_combined	No intervention	0.00	0.00		14023.92	0.00		
Startingcost_combined	NSP	0.00	487.49	484.89:490.09	14023.92	487.49	484.89:490.09	vs no intervention
Startingcost_combined	OST	0.00	1743.40	1734.55:1752.25	14023.92	1743.40	1734.55:1752.25	vs NSP
Startingcost_combined	NSP and OST	0.00	428.49	419.96:436.03	14023.92	461.01	452.98:469.04	vs OST
Startingcost_OST	No intervention	0.00	0.00		58337.53	0.00		
Startingcost_OST	NSP	0.00	487.49	484.89:490.09	58337.53	487.49	484.89:490.09	vs no intervention
Startingcost_OST	OST	0.00	1583.01	1574.15:1591.85	58337.53	1743.40	1734.55:1752.25	vs NSP
Startingcost_OST	NSP and OST	0.00	571.08	562.97:579.03	58337.53	461.01	452.98:469.04	vs OST
**QALYs**								
Variable_NSP	No intervention	0.00	0.00		2.52	0.00		
Variable_NSP	NSP	0.00	50.65	50.2:51.08	2.52	83.21	82.76:83.65	vs no intervention
Variable_NSP	OST	0.00	−2825.37	(−2836.71):(−2793.29)	2.52	−2564.79	(−2585.71):(−2542.29)	vs NSP
Variable_NSP	NSP and OST	0.00	1166.69	1150.94:1181.07	2.52	1086.97	1077.76:1096.24	vs NSP
Variable_OST	No intervention	0.00	0.00		29.20	0.00		
Variable_OST	OST	0.00	33.70	30.63:36.75	29.20	83.21	79.94:86.06	vs no intervention
Variable_OST	NSP	0.00	429.90	419.96:436.03	29.20	−2564.79	(−2585.71):(−2542.29)	vs OST
Variable_OST	NSP and OST	0.00	1086.97	1077.76:1096.24	29.20	1086.97	1077.76:1096.24	vs NSP
Startingcost_combined	No intervention	0.00	0.00		14023.92	0.00		
Startingcost_combined	NSP	0.00	83.21	82.76:83.65	14023.92	83.21	82.76:83.65	vs no intervention
Startingcost_combined	OST	0.00	−2564.79	(−2585.71):(−2542.29)	14023.92	−2564.79	(−2585.71):(−2542.29)	vs NSP
Startingcost_combined	NSP and OST	0.00	1065.64	1049.93:1080.07	14023.92	1086.97	1077.76:1096.24	vs NSP
Startingcost_NSP	No intervention	0.00	0.00		99750.75	0.00		
Startingcost_NSP	NSP	0.00	32.56	29.49:35.62	99750.75	83.21	82.76:83.65	vs no intervention
Startingcost_NSP	OST	0.00	−2970.06	(−2991.71):(−2948.29)	99750.75	−2564.79	(−2585.71):(−2542.29)	vs NSP
Startingcost_NSP	NSP and OST	0.00	1210.95	1194.93:1225.06	99750.75	1086.97	1077.76:1096.24	vs NSP
Startingcost_OST	No intervention	0.00	0.00		58337.53	0.00		
Startingcost_OST	NSP	0.00	83.21	82.76:83.65	58337.53	83.21	82.76:83.65	vs no intervention
Startingcost_OST	OST	0.00	−2328.83	(−2350.54):(−2307.12)	58337.53	−2564.79	(−2585.71):(−2542.29)	vs NSP
Startingcost_OST	NSP and OST	0.00	1086.97	1077.76:1096.24	58337.53	1086.97	1077.76:1096.24	vs NSP

Irrespective of the variation in the starting costs of combined intervention, the results are consistent at both outcome measures. It was found that the variation in the start up costs of combined intervention did not affect the rank of strategies in terms of ICER. Although the ICER of combined intervention varies between $428 and $461/infection averted, the rank of ICER for each intervention did not change. Similarly, when presented in QALYs, the ICER of each intervention did not change regardless of the variation in starting up costs.

On the other hand, it was found that the variable cost of OST can affect the relative rank of strategies. At the lower end of OST, OST was most cost effective strategy at both QALYs and infection averted. The ICER of combined intervention [$428-$461/infection averted] was slightly lower than the ICER of NSP [$487/infection averted] although NSP alone is more cost effective. This results from the fact that OST has ‘extended dominance’ in terms of HIV infections averted and so combined intervention was compared with OST instead of NSP.

In brief, this result relaxes the uncertainty in the start up cost of combined intervention, and offers supporting evidence that the combined intervention and NSP alone are preferred strategies to OST alone. Also, the high variable cost of OST makes OST alone, a less cost effective strategy.

### Probability sensitivity analysis

Figures 2 and 3 shows the results of probabilistic sensitivity analysis with 95% confidence interval using Monte-Carlo simulation.

The result of combined intervention vs NSP alone is located in the first quadrant, suggesting that the combined intervention is incrementally cost effective compared with NSP alone. However, the results indicate that the combined intervention may in fact cost less than OST as a single therapy and therefore be the dominant strategy when compared with OST: 31% of results are in the fourth (south east) quadrant. This means that the probability that the combined intervention is more effective at higher cost, in terms of infection averted, than OST alone is 69% (first quadrant).

Irrespective of the outcome measures, the combined intervention is located below the line of willingness to pay (WTP) for the Ukraine.

## Conclusion

In this paper, an updated systematic review of the cost-effectiveness of harm reduction highlighted a gap in existing evidence: the lack of an incremental approach to comparing the cost effectiveness of combined versus mono-therapy. To fill the evidence gap, this study has attempted to determine the cost-effectiveness of harm reduction by the Global Fund in the Ukraine. A Markov Monte Carlo Model was used to conduct n incremental economic analysis comparing 3 harm-reduction interventions with one another and with no intervention; i.e. NSP alone, OST alone and combined OST and NSP.

The analysis found that all interventions were cost effective in terms of QALYs gained and HIV infections averted. NSP alone was the most cost effective and OST alone was the least cost effective option. While combined therapy did not have the lowest cost effectiveness ratio, it was significantly more effective in both outcome measures than any alternative. The relatively high variable cost of OST ($31.72 per patient per cycle) explains, to a large extent, why the combined strategy is not as cost effective as NSP alone.

The result that NSP is most cost-effective, is consistent with previous research on harm-reduction [19, 25]. NSP alone ($83.35/QALY in Table 4) is shown to be more cost-effective in the Ukraine than in Australia [$416–8,750/QALY] [22] or Kazakhstan [$132–147/QALY] [48], However, the cost effectiveness ratio of NSP alone in this study, at $487.41 per HIV infection averted, is significantly higher than that from another study in the Ukraine study [$97–162 per HIV infection averted] [25]. OST, at $459.90 per QALY, was slightly more cost-effective than other analyses for the Ukraine suggest [$530/QALY] [19]. Regardless, OST was a dominated strategy for both outcome measures and this is attributed to the high variable cost of OST. The results of the sensitivity analysis support this conclusion.

From the result of PSA, the samples are located below the slope of willingness to pay (WTP). Considering that the GDP of the Ukraine was $3,615 in 2011 [34], all three interventions were found to be located below the cost effectiveness threshold suggested by the WHO [35]. As a result, and given the significant dominance of combined therapy in terms of benefits, this is may be the preferred strategy in the Ukraine context. That said, a discussion about affordability and the ethics of selecting a less effective strategy may be warranted in this context.

Some caveats exist in this study and the generalizability of the findings, which should be noted. Firstly, the start-up costs of combined intervention are uncertain. This makes it more important to measure uncertainty and sensitivity in costs. Also, the probability of transmission used in this study assumes normal IDUs. However, depending on the IDUs’ status (i.e. sex workers or men who have sex with men (MSM)), the probability will vary. Likewise, the assumption that the variable cost of the combined strategy is simply the sum of the variable costs for NSP and OST can be relaxed with more detailed information regarding cost data. Another caution is that the effectiveness parameters, such as drop-out rates, will vary depending on how an intervention is implemented in practice. Therefore, the ‘dominance’ of each strategy over comparators should be carefully interpreted. Also, the GDP threshold is a weak indicator of affordability and more work is needed in this area.

More detail on the number of IDUs reached by Global Fund NSP and OST programs would further improve the accuracy of estimates. With more evidence regarding the effectiveness of harm reduction programs, including those supported by the Global Fund, greater support for effective – and cost effective - harm reduction can be fostered.

## Abbreviations

NSP: Needle and syringe programme
OST: Opioid substitution therapy
ART: Anti-retroviral therapy
IDUs: Injecting drug users.
